# Accumulation of amyloid beta (Aβ) and amyloid precursor protein (APP) in tumors formed by a mouse xenograft model of inflammatory breast cancer

**DOI:** 10.1002/2211-5463.13308

**Published:** 2021-10-26

**Authors:** Astrid Zayas‐Santiago, Michelle M. Martínez‐Montemayor, Jadier Colón‐Vázquez, Gabriela Ortiz‐Soto, Jose G. Cirino‐Simonet, Mikhail Inyushin

**Affiliations:** ^1^ Department of Physiology Universidad Central del Caribe Bayamón PR USA; ^2^ Department of Biochemistry Universidad Central del Caribe Bayamón PR USA

**Keywords:** inflammatory breast cancer, xenografts, tumor, amyloid beta, immunostaining

## Abstract

Accumulation of amyloid in breast cancer is a well‐known phenomenon, but only immunoglobulin light‐chain amyloidosis (AL) or transthyretin (TTR) amyloid had been detected in human breast tumor samples previously. We recently reported that another amyloidogenic peptide, amyloid beta (Aβ), is present in an aggregated form in animal and human high‐grade gliomas and suggested that it originates systemically from the blood, possibly generated by platelets. To study whether breast cancers are also associated with these Aβ peptides and in what form, we used a nude mouse model inoculated with triple‐negative inflammatory breast cancer cell (SUM‐149) xenografts, which develop noticeable tumors. Immunostaining with two types of specific antibodies for Aβ identified the clear presence of Aβ peptides associated with (a) carcinoma cells and (b) extracellular aggregated amyloid (also revealed by Congo red and thioflavin S staining). Aβ peptides, in both cells and in aggregated amyloid, were distributed in clear gradients, with maximum levels near blood vessels. We detected significant presence of amyloid precursor protein (APP) in the walls of blood vessels of tumor samples, as well as in carcinoma cells. Finally, we used ELISA to confirm the presence of elevated levels of mouse‐generated Aβ40 in tumors. We conclude that Aβ in inflammatory breast cancer tumors, at least in a mouse model, is always present and is concentrated near blood vessels. We also discuss here the possible pathways of Aβ accumulation in tumors and whether this phenomenon could represent the specific signature of high‐grade cancers.

## Graphical abstract

Aβ (amyloid beta) and it's precursor APP can be observed in tumors formed by mouse xenograft model of human inflammatory breast cancer (IBC). Aβ is present in and around carcinoma cells and is concentrated near blood vessels. We suggest that systemic Aβ from mouse blood and/or vessels (containing amyloid precursor APP) may be, jointly with other amyloidogenic peptides, forming aggregated amyloid in these tumors.

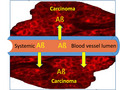

**Abbreviations**
AD: Alzheimer’s disease
AL: light‐chain amyloidosis
APP: amyloid precursor protein
Aβ: amyloid beta
DAPI‐4′: 6‐diamidino‐2‐phenylindole
ELISA: enzyme‐linked immunosorbent assay
HER2/neu: human epidermal growth factor receptor 2 (proto‐oncogene)
IBC: inflammatory breast cancer; SUM‐149‐IBC cell line
MALT: mucosa‐associated lymphoid tissue
MAPK: mitogen‐activated protein kinases
PDGF: platelet‐derived growth factor
SCID: severe combined immunodeficient (mice strain)
TNM: tumor–nodes–metastasis classification of cancers
TTR: transthyretin

Inflammatory breast cancer (IBC) is one of the most aggressive types of this disease; it is highly metastatic and usually fatal (as it is commonly diagnosed at T4, according to the tumor–nodes–metastasis classification of cancers (TNM) classification). Various IBC carcinoma cell subtypes exist, with the triple‐negative subtype the most frequently diagnosed. These tumors lack receptors for estrogen, progesterone, or human epidermal growth factor receptor 2 (proto‐oncogene) (HER2/neu), and thus, IBC is often treated with chemotherapy, radiation, or surgery, due to the lack of targeted therapies. While IBC incidence is about 2%–5% of all breast cancers (with 70% higher occurrence in African American and younger women), it develops rapidly, accounting for 10% of breast cancer deaths annually in the United States [1, 2]. Histologically, IBC has specific features of inflammation, with pathological evidence of cancer that includes increased angiogenesis and high infiltration of tumor‐associated (immune‐suppressing) macrophages involving tumor emboli in dermal‐lymphatic vessels by large clusters of circulating tumor cells [3, 4]. It was found that IBC‐type carcinoma cells produce elevated levels of vascular endothelial growth factor, combining angiogenesis with enhanced vessel porosity [5]. Studying the expression of proteins important for breast cancer diagnostics in order to find specific markers of IBC enabled the discovery of elevated E‐cadherin [6] and P‐cadherin (cadherin signaling pathways control invasion and metastasis) expression in IBC tumors [7], but they are present in some non‐IBC tumors as well. Besides cadherin, no unique genomic marker has been conclusively identified for IBC; thus, no specific molecular therapeutic approaches have yet been proposed to manage IBC [8]. Therefore, finding specific markers for high‐grade breast cancers, such as IBC, became a recognized priority.

Recently, statistically independent cohort studies have found an inverse association between cancers in general (including breast cancer) and Alzheimer’s disease (AD; [9, 10, 11, 12, 13]). These findings suggest that there are common factors in these diseases. Possible linkages in this cancer–AD relationship have been proposed: decreased mitochondrial metabolism, in general, and decreased signaling through the p53, Pin1, and Wnt cellular signaling pathways, in particular [9, 14]. Besides these, the buildup of amyloid precursor protein (APP), the precursor of the AD hallmark amyloid beta (Aβ) peptides, has been discovered in breast cancer tumors and corresponding metastatic lymph nodes [15]. Initially, it was found that the proteolytic cleavage of APP by the α‐secretase (non‐amyloid) pathway reduces proliferation and migration in breast cancer [16]. Later, it was confirmed that APP controls breast cancer through the mitogen‐activated protein kinases (MAPK) signaling pathway, regulating different kinases and the expression of filaments such as cadherins, cytokeratins, and vimentin. Generally, it was shown that APP overexpression increases the migratory and invasive ability of human breast cancer cells, whereas APP silencing significantly inhibits cell migration and invasion [17]. It was also discovered that APP has differential expression in different breast cancer cell lines, with triple‐negative breast cancer cell lines having the highest level of APP expression [16]. We found no published evidence on whether APP is cleaved to form Aβ peptides in breast cancer.

It was reported that Aβ peptide levels in blood plasma are significantly elevated in esophageal cancer, colorectal cancer, hepatic cancer, and lung cancer patients as well as in melanoma and adenocarcinoma [18, 19, 20]. We recently reported that Aβ is expressed in glioma cells and in close proximity to blood vessels in mouse and human late‐stage glioma tumors [21, 22]. Do IBC tumors also contain Aβ peptides, what is the source of this Aβ, and in what form does it take in IBC?

While it is known that APP is present in IBC, we found no published evidence on whether APP is cleaved to form Aβ peptides and aggregated Aβ amyloid in breast cancer. However, besides carcinoma cells themselves, another blood‐related systemic source of Aβ peptide production may lead to its accumulation in tumors [23, 24]. Recently, we found that platelets trigger a massive release of Aβ after thrombosis and that this release is concentrated near blood vessels [25, 26]. It is known that platelets in cancer patients are hyperactivated and form cancer cell‐induced aggregates and micro‐thrombi in the vasculature near tumors. Poor survival in a large variety of cancers, including metastatic breast cancer, is associated with high platelet count, with thrombocytopenia or antiplatelet drugs reducing the short‐term risk of cancer, cancer mortality, and metastasis [27]. Thus, platelet count may be a prognostic marker for breast cancer [28, 29]. Previously, it was suggested that platelets affect cancer cells by releasing platelet‐derived growth factor (PDGF; [30]). However, it was recently shown that Aβ oligomers inhibit the growth of various human cancer cells, including breast carcinoma cells [31]. Thus, there is the possibility that platelet‐generated Aβ is produced in response to cancer development and released in tumors, as we suggested previously [24].

It is known that rarely (only in 7% of cases) aggregated amyloid, predominantly light‐chain amyloidosis (AL) or sometimes misfolded transthyretin (TTR)‐type amyloid, are detected in human breast tumor samples [32, 33, 34, 35, 36, 37]. In patients with breast cancer and AL‐type amyloid, over half have concurrent breast hematologic disorders [35, 38].

In our study, we chose specific antibodies against Aβ peptides with low reactivity for its precursor APP to see whether Aβ immunoreactivity is present in xenograft tumors induced by triple‐negative inflammatory breast cancer cells. In addition, the presence of APP itself was assessed in the same tumor tissues in general as well as in tumor blood vessels. The presence of aggregated forms of amyloid inside tumors was evaluated using fluorescence histochemistry staining with dyes selective for aggregated β‐pleated sheet amyloid. We also assessed whether Aβ immunoreactivity and aggregated amyloid are concentrated near blood vessels.

## Results

### Immunoreactivity against Aβ peptides and APP is present in all studied tumor samples but not in control skin samples

**Fig. 1:**
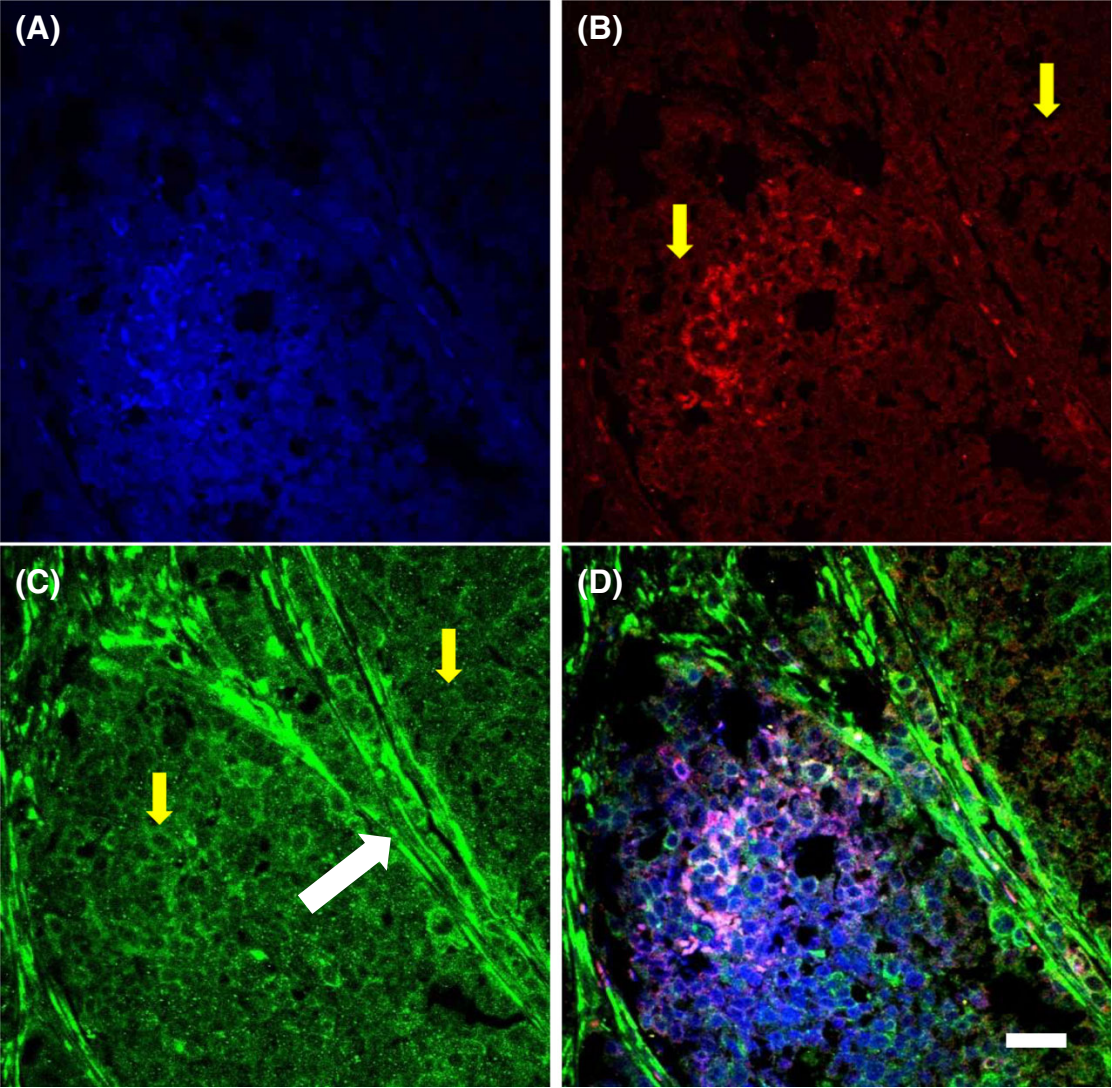
Carcinoma cells have both Aβ and APP immunoreactivity. (A) DAPI (blue); (B) Aβ immunoreactivity (MOAB‐2, red); (C) APP immunoreactivity (green); (D) A + B + C (overlay). Scale bar, 20 µm; arrows, see text.

Triple‐negative inflammatory breast cancer cell (SUM 149) tumors that were developed over 10 weeks after xenograft subcutaneous inoculations of hairless severe combined immunodeficient (SCID) mice were harvested, as described previously [39]. A total of three tumors from different animals were sliced and analyzed. Applying immunocytochemistry, we found that all tumor samples had both APP and Aβ staining in a majority of the carcinoma cells (Fig. 1B,C, yellow arrows). It was also found that APP is specifically concentrated in the endothelial cells of blood vessels (Fig. 1C, white arrow). Interestingly, the Aβ‐specific antibody MOAB‐2, reactive against Aβ residues 1–4 and which recognizes both murine and human Aβ peptide, stained not only carcinoma cells and some endothelial cells but also extracellular amyloid, most probably in fibrillary or aggregated form (Fig. 1B). The same amyloid was stained with 4′,6‐diamidino‐2‐phenylindole (DAPI, Fig. 1A), which is a fluorescent dye that binds to DNA and normally stains only the cell nucleus at visible levels. It was reported previously that DAPI specifically stains amyloid light‐chain (AL) aggregates (while not labeling type A amyloid [AA]; [40]). Similarly, DAPI was reported to stain Aβ plaques as well, although this finding was unpublished (ResearchGate, 2014) [41]. Thus, aggregated amyloid in xenograft tumors is either (a) pure Aβ amyloid or (b) composed of mixed amyloids containing both Aβ (stained with MOAB‐2) and AL aggregates (stained with DAPI).

Control staining of healthy skin shows no visible presence of Aβ and APP under the same recording conditions (Fig. S1).

### Specific histochemical staining against β‐sheet amyloid was present in all studied tumor samples but not in control skin samples

**Fig. 2:**
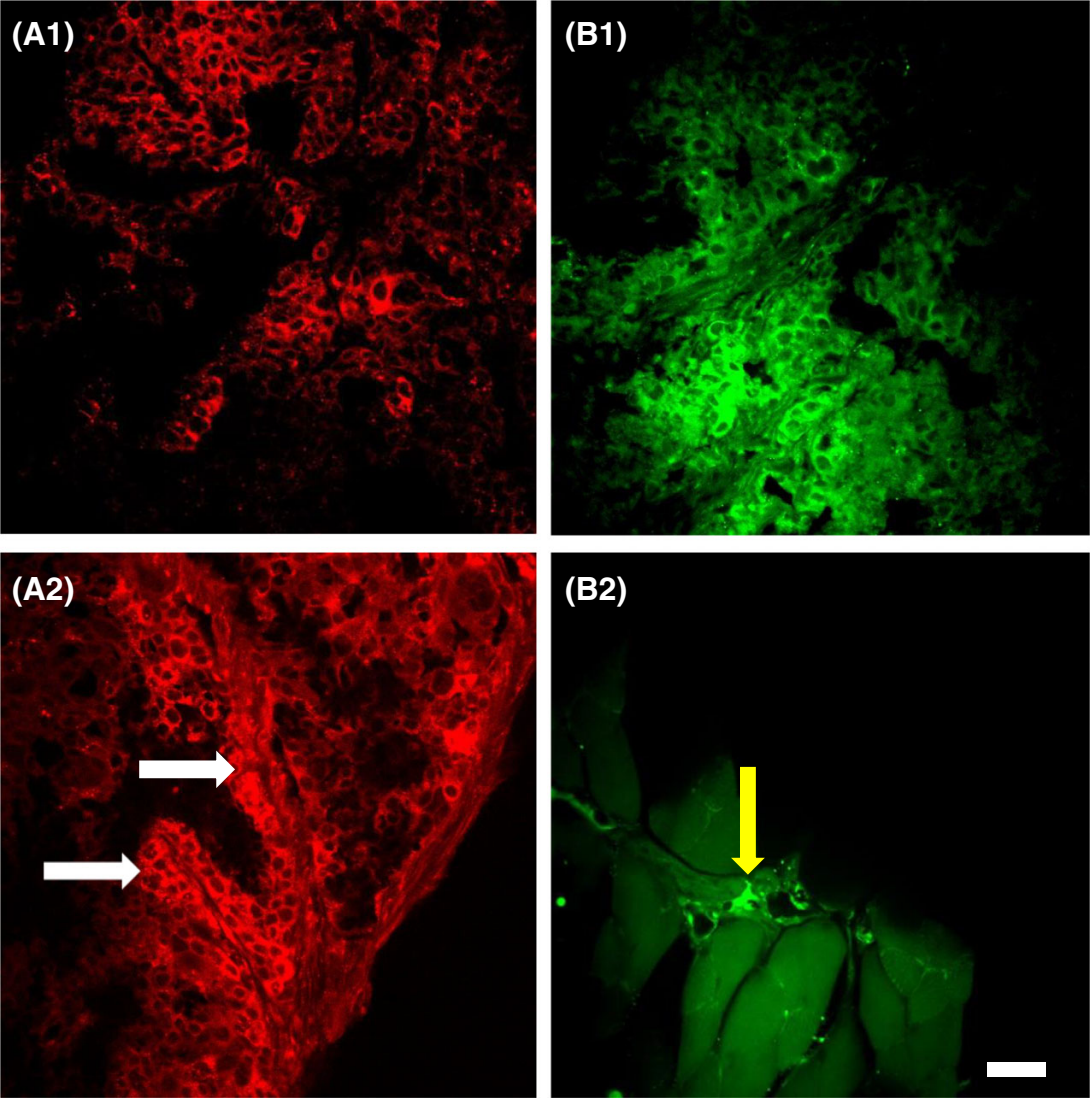
Carcinoma stained with Congo red (A1, A2, red) and thioflavin S (B1, B2, green). Some zones have enhanced fluorescence (white arrows). Carcinoma cells in adipose tissue (yellow arrow). Scale bar, 20 µm.

Slices from the same tumor samples were stained using Congo red and thioflavin S, both dyes known as specific fluorescent stains for aggregated β‐pleated sheet amyloid [22, 42, 43, 44, 45]. Both Congo red (Fig. 2A1, A2) and thioflavin S (Fig. 2B1, B2) clearly stained carcinoma cells, which are recognizable because of their relatively similar shape and size. In addition, in adipose tissue attached to the tumor, some carcinoma cells fluorescently stained with thioflavin S can be seen around blood vessels (Fig. 2B2, yellow arrow). While the majority of carcinoma cells were stained with dyes, the staining was not uniform. Certain zones were obviously stained with slightly enhanced fluorescence (Fig. 2A2, B1, white arrows). We interpreted these enhancements as additional aggregated extracellular amyloid, but it may also be that in some carcinoma cells amyloid is concentrated. Similarly, the immunostaining of tumor carcinoma cells was also not uniform (Fig. 1). Previously, in studying glioma tumors we found that cells near blood vessels had enhanced amounts of Aβ [21]. We hypothesized that amyloid is concentrated in specific places because of nearby blood vessels and that blood is the source of Aβ (at least partially). Therefore, we analyzed the corresponding confocal brightfield images of blood vessels.

### Specific staining with Aβ amyloid antibodies (staining all forms of Aβ) and with Congo red/thioflavin S (against β‐pleated sheet Aβ) is concentrated near blood vessels

**Fig. 3:**
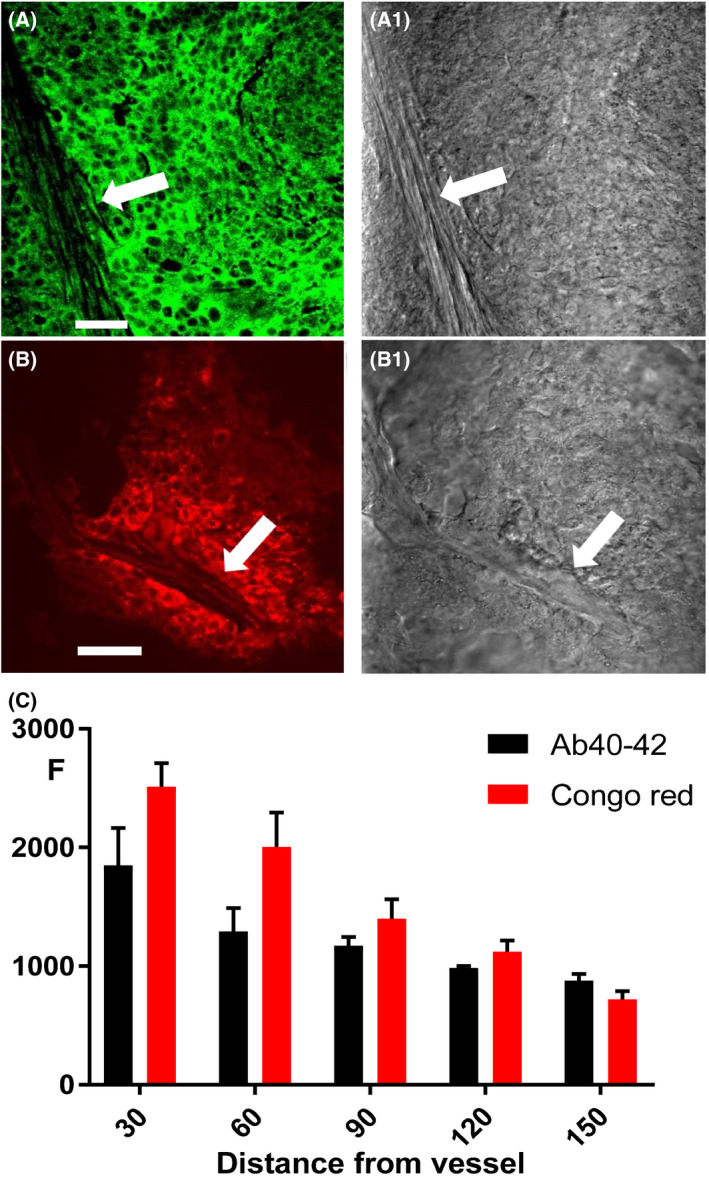
Tumor tissue stained with a Aβ1–42 polyclonal antibody reactive with Aβ40 and 42 (A, green) and Congo red (B, red). The corresponding confocal brightfield images (A1 and B1) outline blood vessel boundaries (white arrows). A plot (C) shows the fluorescence level (F) at different distances (in µm) from blood vessels; error bars represent SD. Scale bar, 25 µm.

Brightfield images clearly indicated blood vessel boundaries (Fig. 3A1, B1, white arrows) allowing us to measure the distance from the blood vessel to a tissue. We used corresponding fluorescence images of stained tissue, either with antibodies against Aβ or with a histochemical stain against β‐pleated sheet amyloid (example shown in Fig. 3A,B). imagej analysis software (https://imagej.nih.gov/ij/) was applied, allowing us to measure the mean fluorescence level at particular loci at various distances from nearby blood vessels. This procedure allowed us to construct a graph of the dependence of Aβ‐related fluorescence (F, in arbitrary units) on the distance from blood vessels (in µm; Fig. 3C). It was confirmed that Aβ immunofluorescence is concentrated near blood vessels. A total of five slides with Congo red and Aβ40–42 staining were analyzed, and the values near blood vessels appeared to be greater than at a distance of 30 µm or more (*t*‐test: *t* = 9.535, df = 8, *P* < 0.001 for Congo red; *t* = 3.216, df = 8, *P* = 0.012 for Aβ40–42). It was then determined that levels of Aβ immunofluorescence and β‐pleated sheet amyloid staining were effectively correlated (correlation coefficient 0.9549, *P* < 0.01). Thus, we confirmed that blood vessels are important for Aβ distribution in breast cancer tissue (see also the S3 video). Interestingly, DAPI staining was also concentrated in the vicinity of blood vessels (Fig. S2). We ascribe this to the propensity of DAPI to stain aggregated amyloid.

### Murine Aβ40 is elevated in studied tumor samples

In our histochemical studies, we used pan‐specific antibodies recognizing Aβ peptides from different species. Because Aβ in xenograft tumors may have both a murine (systemic) and/or human (produced by human cancer cells) origin, we used a commercially available mouse‐specific enzyme‐linked immunosorbent assay (ELISA) kit to see whether systemic mouse Aβ40 is concentrated in tumors. According to the manufacturer, this mouse Aβ40 ELISA kit is very specific, and mouse Aβ40 had no cross‐reactivity with human Aβ40 even till 10 ng·mL^−1^. This allowed us to compare the freshly frozen samples from the xenograft tumors and controls. Measurements indicate that mean concentrations of mouse Aβ40 peptides in tumors (1240 ± 149.8, pg·mL^−1^) versus controls (316.7 ± 14.53, pg·mL^−1^) are increased about fourfold (*P* < 0.05, *t* = 6.136, df = 4).

## Discussion

We used two different specific antibodies against to visualize Aβ peptide distribution in inflammatory breast cancer SCID mouse model xenografts. It was found that both (a) MOAB‐2 monoclonal antibodies specific for residues 1–4 of Aβ (with low affinity for APP) and (b) Aβ1–42 polyclonal antibodies specific for the amino acid sequence at Aβ40–42 specifically stain carcinoma cells (Figs 1B and 3A). Additionally, some aggregated forms of amyloid were stained. MOAB‐2 antibodies stained these aggregates or filaments, but they were outlined by DAPI as well (Fig. 1A,B,D). It was reported previously that DAPI specifically stains amyloid light chain (AL) aggregates (while not staining AA; [40]). In addition, DAPI was described to stain Aβ plaques as well, although these results were not published [41]. Because there is no published evidence of DAPI staining of Aβ aggregates, there are two possibilities; aggregated amyloid in xenograft tumors is (a) pure Aβ amyloid or (b) a mixture of amyloid containing both Aβ (stained with MOAB‐2) and AL aggregates (stained with DAPI).

Amyloid aggregates were also detected using fluorescence histochemical methods. We used two different amyloid‐specific fluorescent dyes (Congo red and thioflavin S). Fluorescence detection of samples allows improved detection of amyloid in cytology preparations [45, 46, 47].

Using both dyes, we found intensive staining of amyloid in an aggregated form, especially near blood vessels (Figs 2A, panels A1 and B1, also 3B and S3 GIF video). While we also detected aggregated amyloid with Aβ immunostaining (Fig. 1), it is uncertain whether it is the only component of these aggregates.

Previous reports have established that aggregated amyloid in the breast is mainly of the AL type (usually κ light chain); in half of patients, it is part of an additional systemic immune cell disease, and in 55% of patients, it is associated with a hematologic malignancy of the breast (usually mucosa‐associated lymphoid tissue (MALT) lymphoma; [35, 36]). By definition, L‐amyloidosis is produced by the kappa light chain of immunoglobulin M (IgM). However, there is no literature investigating L‐type amyloid in nude SCID mice. While the nude mouse model that we used is immunocompromised, IgM levels are usually unchanged (unlike other immunoglobulins) and similar to the levels of IgM in wild‐type mice [48]. This finding suggests that L‐type amyloid may be present in this mouse strain as well. Thus, amyloid in xenograft tumors may be of mixed type, and this question needs further study.

Carcinoma cells were also stained with antibodies against different isoforms of APP, but most distinctively anti‐APP antibody marked blood vessel walls within the tumor (Fig. 1C,D). The antibody we used does not specifically recognize species‐specific APP, and the origin of APP in xenograft tumors is not well established. It was shown that the stroma and vessels supporting the growth of many types of xenograft tumors were of murine origin or possibly a mosaic [49, 50, 51]. It is known that human and murine APP have only 86% similarity, and specific antibodies can detect the difference [52, 53]. To prove that Aβ within the tumor has at least a partial murine origin, we used mouse‐specific ELISA to measure Aβ in tumors and controls. It was found that the concentration of mouse‐originated Aβ40 is significantly elevated (~ 4‐fold) in tumors, which confirmed the possible systemic mouse origin of amyloid. Thus, the APP that we observed in tumor blood vessels may be of murine origin, while the carcinoma cells that were injected to form xenografts were of human origin. Of course, future specific experiments will show whether human APP and Aβ are also present in tumors and determine the presence or absence of other amyloidogenic peptides (including possibly AL). Also, we confirmed that visible amounts of the Aβ precursor APP are present in xenografts tumors. While APP buildup has been reported previously in breast cancer tumors and the corresponding metastatic lymph nodes [15], the presence of its β‐secretase cleavage product, Aβ, was not reported. Moreover, according to the literature, APP has shown differential expression in different breast cancer cell lines. It was found that triple‐negative breast cancer cell lines have the highest levels of APP expression [16]. These authors also discovered that if the proteolytic cleavage of APP is processed by α‐secretase (the non‐amyloid pathway), proliferation and migration in breast cancer are reduced [16]. It was found that APP overexpression increased the migratory and invasive ability of human breast cancer cells, whereas APP silencing significantly inhibited cell migration and invasion [17]. Based on the results presented here and in the literature, we suggest that APP processing in the β‐secretase pathway would augment cell migration. The implication is that Aβ production indicates only highly invasive breast cancers, such as IBC.

There have been speculations that APP affects carcinoma cell mobility through APP binding protein 2 (APPBP2), which is highly expressed in breast cancer (https://www.genecards.org/cgi‐bin/carddisp.pl?gene=APPBP2). Unfortunately, the expression of this protein concerning invasive properties is not known.

We still do not know whether carcinoma cells themselves process APP to generate Aβ. We have discovered that both Aβ immunoreactivity and aggregated amyloid are concentrated near blood vessels (Fig. 3), suggesting that blood vessels are important for Aβ production. In addition, Aβ of murine origin is elevated in tumors, demonstrating the murine and probably blood‐related source of these peptides. These results suggest that systemic production of Aβ is important for its accumulation in tumors, including in the aggregated form, although other sources may also be involved, including carcinoma cells themselves, since in a previous study we found that mouse Aβ alone does not produce aggregated amyloid [53]. We also found a similar Aβ accumulation in aggressive gliomas [21, 22], which could represent a specific signature of high‐grade cancers. One future direction will be the study of Aβ production by inflammatory breast cancer cells in vitro. Another important task will be to determine whether Aβ production in this kind of tumor can be regulated to inhibit growth and migration.

## Conclusions

We confirmed that Aβ peptides in inflammatory breast cancer tumor xenografts are always present in visible amounts and are concentrated near blood vessels within the tumors. The tumors studied also showed a strong presence of aggregated amyloid containing Aβ. While we studied only a xenograft model of IBC, we suggest that Aβ is present in inflammatory breast cancers in general and may be used as a marker of these cancers, but this possibility must be further evaluated.

## Materials and methods

### Cell culture and mouse subcutaneous SUM‐149 cell tumors

#### Cell culture

The patient‐derived IBC cell line SUM‐149 (BioIVT, Westbury, NY, USA) was cultured in Ham’s F12 medium (Life Technologies, Carlsbad, CA, USA) with 10% fetal bovine serum (FBS), as previously described [54, 55, 56]. SUM‐149 IBC cells were seeded 1 : 3 in 10‐cm plates. Once they reached 70% confluency, 3 mL of culture media was collected every 72 h from three plates, while regular media served as control. For total protein extraction, the cells were harvested with a cell scraper and lysed on ice using NP‐40 lysis buffer containing one protease inhibitor tablet (Roche Diagnostics Corporation, Indianapolis, IN, USA). Cells were incubated for 10 min on ice and vortexed intermittently. Supernatants were collected after centrifugation [21,256 ***g*** (RCF), 4 °C, 10 min], and protein concentration was quantified using the Precision Red protein assay kit (Cytoskeleton, Inc., Denver, CO, USA), as described previously [54, 56, 57].

#### Mouse subcutaneous tumors

Female severe combined immunodeficient (SCID) mice (21 days of age) were purchased from Charles River Laboratories International Inc. (Wilmington, MA, USA) and housed under specific pathogen‐free conditions. The mice received an autoclaved AIN 76‐A phytoestrogen‐free diet (Tek Global, Harlan Teklad, Madison, WI, USA) and water *ad libitum*. Cell inoculations were performed as previously described by us [39, 56]. Briefly, SUM‐149 cells (3.0 × 10^6^, 1 : 1 serum medium: Matrigel; BD Biosciences, San Jose, CA, USA) were injected into the fourth right mammary fat pad under isoflurane inhalation. Mice were weighed weekly, while tumor weights were obtained at the end of the study (10 weeks). This project was approved by the UCC Institutional Animal Care & Use Committee (IACUC, protocol #037‐2021‐15‐01‐PHA‐IBC).

### Tissue preparation and cryosectioning

Tumors were excised and fixed in a solution of 4% paraformaldehyde in phosphate‐buffered solution (PBS: NaCl, 137 mm; KCl, 2.7 mm; Na2HPO4, 10 mm; KH_2_PO_4_, 1.8 mm; in distilled water at pH 7.4) and incubated overnight (16–24 h) in the same fixative solution. After incubation, the tissues were transferred to PBS. After rinsing the tissues three times for 10 min per rinse at room temperature (RT) in 5% sucrose in PBS with gentle rotation, the tissues were cryoprotected with increasing concentrations of sucrose in PBS, which were obtained by mixing 5% sucrose and 20% sucrose in ratios of 2 : 1, 1 : 1, and 1 : 2. For each ascending concentration of sucrose, the tissues were incubated for 30 min at RT, and the tissues were then incubated overnight first in 20% sucrose and then in 30% sucrose at 4 °C. After this step, the samples were stored at −80 °C or immediately prepared for cryosectioning. Tissues were sectioned at a thickness of 30 µm using a cryostat (CM1860, Leica Biosystems, Wetzlar, Germany) at −23 °C.

### Immunostaining, histochemistry, and fluorescence confocal microscopy

The slides were air‐dried for 30 min and immunostained using a previously established protocol [22, 58]. First, sections were treated for 20 min with a permeabilization solution consisting of 0.03% Triton X‐100 (Sigma‐Aldrich, St. Louis, MO, USA) and 1% dimethyl sulfoxide (DMSO; MP Biomedicals, Santa Ana, CA, USA) in PBS under gentle agitation (40 rpm). Second, the sections were treated for 60 min with a blocking solution containing either 5% normal goat serum, 5% normal horse serum, or 5% rabbit normal serum (Vector Laboratories, Burlingame, CA, USA) and 2% w/v bovine serum albumin (BSA; Sigma‐Aldrich) in the permeabilization solution. Following the blocking step, the sections were processed with antibodies against Aβ and APP. Slices were incubated with a mouse monoclonal antibody to Aβ (MOAB‐2, 1 : 200; Abcam, Cambridge, MA, USA, cat. #ab126649), a rabbit polyclonal antibody to Aβ1–42 (1 : 200; Abcam, cat. #ab216504), and a rabbit polyclonal antibody to APP (1 : 200; Abcam, cat. #ab15272), all incubated overnight at 4 °C. After three washes with permeabilization solution for 10 min, the samples were incubated with either secondary goat antibody to rabbit IgG conjugated to Alexa Fluor® 647 (1 : 400; Abcam, cat. #ab150083), secondary horse antibody to mouse IgG conjugated to Texas red® (1 : 400; Vector Labs, TI‐2000), or secondary rabbit antibody to goat IgG conjugated to FITC (1 : 400; Vector Labs, FI‐5000) with shaking for 2 h at room temperature and protected from light. The slices were then washed three times with PBS for 10 min and once with distilled water before being transferred onto a glass slide containing Fluoroshield™ mounting medium (Sigma‐Aldrich, cat. #F6057) with DAPI.

For thioflavin staining, we used thioflavin S (Th‐S). Human brain slices (30 µm) containing tumors were completely air‐dried before staining. The slides were washed twice with 450 µL of 100% EtOH for 2 min each wash. Next, the slides were washed once with 450 µL of 70% EtOH for 2 min then washed once with 450 µL of 80% EtOH for 2 min. Finally, the slides were washed with 450 µL of tap water, 450 µL of distilled water, and 450 µL of alkaline alcohol solution for 2 min each, then incubated for 5 min with Th‐S. After incubation, the slides were washed with 450 µL of tap water, then dehydrated with 450 µL of 95% EtOH for 3 min and two changes of 450 µL of 100% EtOH for 3 min each. The coverslip was mounted with a drop of Vectashield® mounting medium to visualize fluorescence (Vector Laboratories, Burlingame, CA, USA, cat. #H‐1000).

Congo red staining was performed as follows. A 0.5% Congo red solution was prepared in 50% alcohol. Human brain slices (30 µm) containing tumors were air‐dried thoroughly and then washed twice with absolute alcohol for 2 min. The slides were washed twice with 450 µL of 100% EtOH for 2 min each wash, followed by washing once with 450 µL of 70% EtOH for 2 min, 450 µL of 80% EtOH for 2 min, and 450 μL of tap water and incubated for 20 min in a filtered solution of Congo red (0.2‐µm filter). After incubation, the slides were rinsed once with distilled water, incubated in 450 µL of alkaline alcohol solution for 2 min, and then washed with 450 µL of tap water. Finally, the slices were dehydrated with 450 µL of 95% EtOH for 3 min, with two changes of 450 µL of 100% EtOH for 3 min each. The glass slides were mounted with Fluoroshield mounting medium containing 4′,6‐diamidino‐2‐phenylindole (DAPI).

FITC excitation/emission filters were used to visualize specific amyloid‐associated Th‐S and a Texas‐Red filter for Congo red fluorescence.

Images were acquired using an Olympus Fluoview FV1000 scanning inverted confocal microscope system equipped with 4x, 10x, 20x, or 40x/1.43 oil objectives (Olympus, Tokyo, Japan). The images were analyzed using imagej software (ver. 1.8.0_112, http://imagej.nih.gov/ij) with the Open Microscopy Environment Bio‐Formats library and plugin (http://www.openmicroscopy.org/site/support/bio‐formats5.4/), allowing for the opening of Olympus files. The images were evaluated using custom colorization.

### Enzyme‐linked immunosorbent assay measurements

A specialized, ready‐to‐use, mouse‐specific, solid‐phase sandwich ELISA kit (cat. #KMB3481; Invitrogen, Thermo Fisher Scientific, Waltham, MA, USA) was used for direct measurement of the amount of mouse Aβ40 peptide in tumors and controls, as we described previously (Kucheryavykh *et al*., 2019) [21]. The tissue samples were homogenized mechanically, and 100 mg of homogenate from each sample was then lysed in guanidine solution (5 M guanidine HCl, 50 mm Tris/HCl, pH 8.0). A monoclonal antibody against the NH_2_ terminus of mouse Aβ40 was coated onto the wells of the microtiter strips provided in the kit. Samples, including standards of known Aβ40 content for calibration purposes as well as experimental specimens, were pipetted into the wells. After washing, the rabbit antibody specific to the COOH terminus of Aβ40 was added and detected with horseradish peroxidase‐labeled anti‐rabbit antibody. The optical density values at 450 nm were determined using a Wallac 1420 Victor2 microplate reader (PerkinElmer Inc., Waltham, MA, USA). A standard curve was used for final determination of the concentration of Aβ40 in the samples and is presented as picograms of Aβ40 per milliliter of initial homogenate.

### Statistics and measurements

Using graphpad prism 7.03 (GraphPad Software, Inc., La Jolla, CA, USA) for calculations, an unpaired *t*‐test was employed to estimate statistical differences. Values were determined to be significantly different if the two‐tailed *P*‐value was < 0.05.

## Conflict of interest

The authors declare no conflict of interest.

## Author contributions

MI, MM, and AZ conceptualized the study; AZ, MM, JC‐V, JC‐S, and GS contributed to methodology; MM, MI, and AZ validated the data; AZ made formal analysis; JC‐V, JC‐S, and GS contributed to data curation; MI involved in initial draft preparation; AZ, MM, MI, JC‐V, JC‐S, and GS reviewed and edited the manuscript; MM and AZ supervised the study; AZ, MM, and MI involved in project administration. All authors have read and agreed to the published version of the manuscript.

## Supporting information


**Fig. S1.** (A) Ab1‐40‐control tissue; (B) MOAB‐2 ‐control tissue; (C) Tioflavin S ‐ control tissue; (D) Congo Red (red) ‐ control tissue, Scale bar 20 µm.

**Fig. S2.** DAPI staining of amyloid near blood vessels, Scale bar, 20 µm.

**Fig. S3.** GIF video in which confocal Congo red fluorescence is concentrated near blood vessels. Original files from confocal microscope in TIFF format are present as zipped Supplementary Information files.

## Data Availability

The data that support the findings of this study are available in the supplementary material of this article.
