# Supplementary figures and images for: Accumulation of amyloid beta (Aβ) and amyloid precursor protein (APP) in tumors formed by a mouse xenograft model of inflammatory breast cancer
Source: FEBS Open Bio. 2021 Oct 26;12(1):95–105. doi: 10.1002/2211-5463.13308 (PMC8727955; doi:10.1002/2211-5463.13308)

## Slide 1
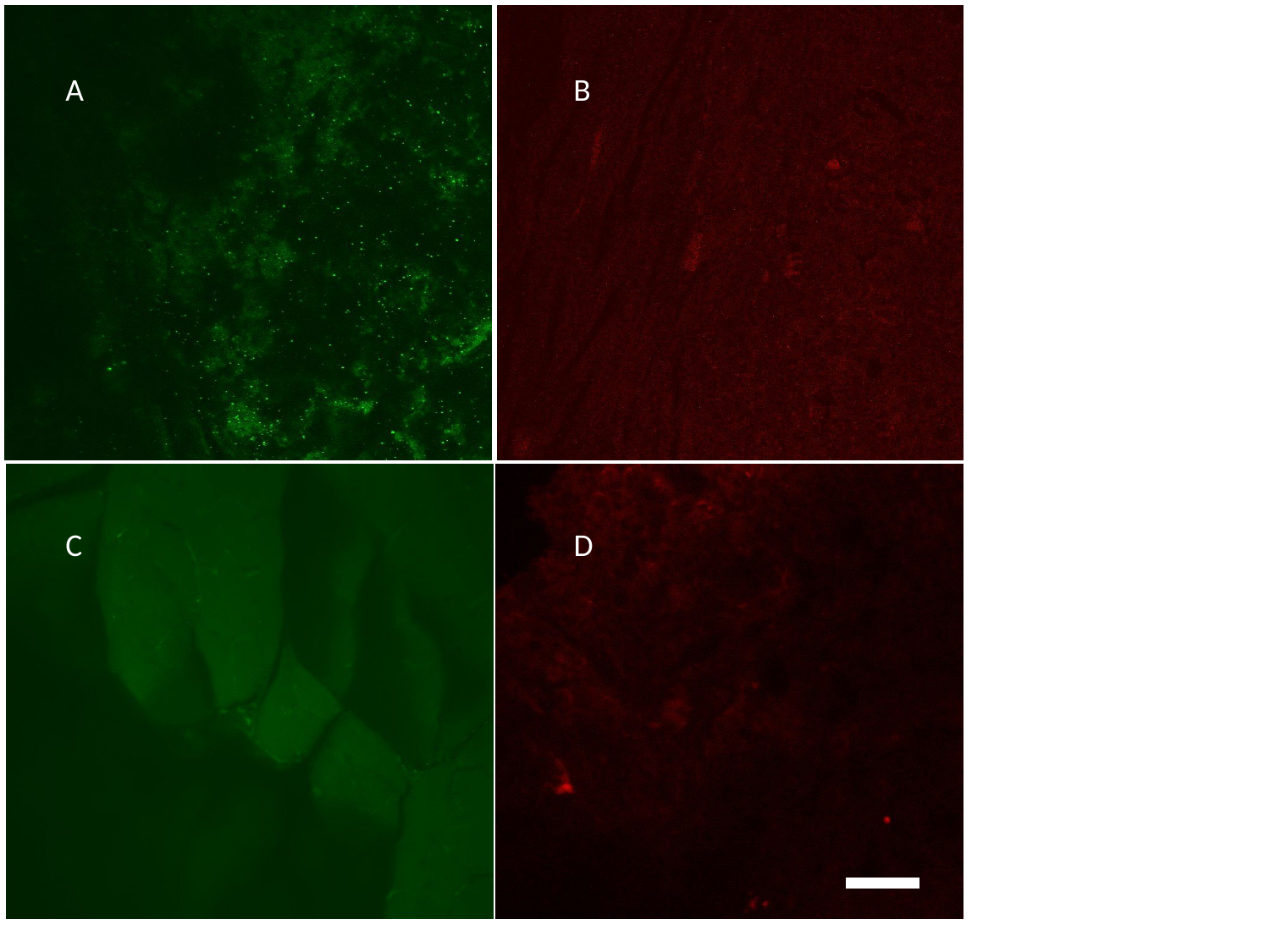

A
B
C
D

Supplement: Supplementary file 1 — Fig. S1. (A) Ab1‐40‐control tissue; (B) MOAB‐2 ‐control tissue; (C) Tioflavin S ‐ control tissue; (D) Congo Red (red) ‐ control tissue, Scale bar 20 µm. [file FEB4-12-95-s002.pptx]

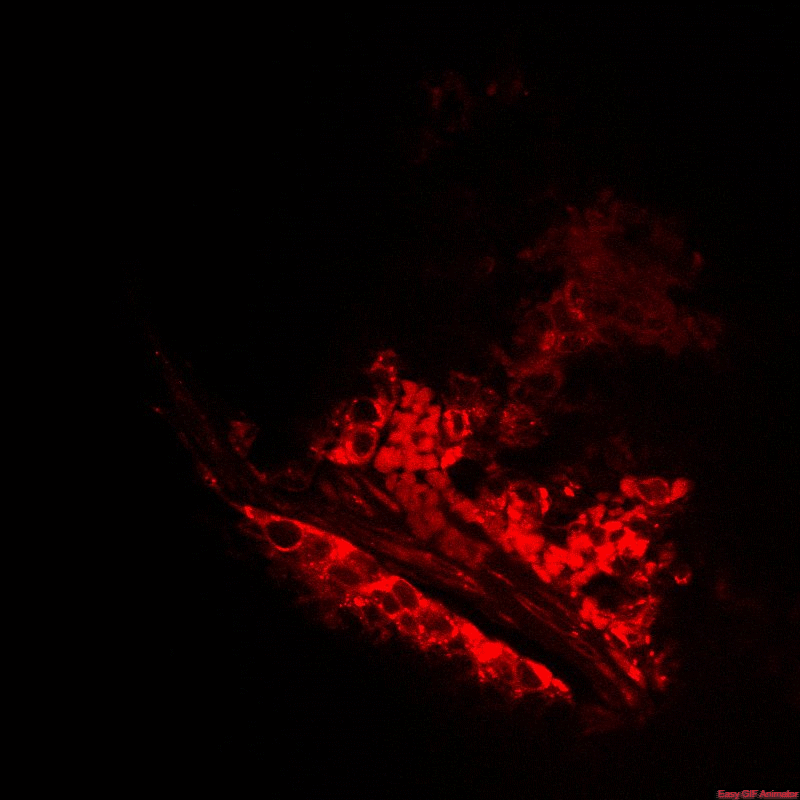

Supplement: Supplementary file 3 — Fig. S3. GIF video in which confocal Congo red fluorescence is concentrated near blood vessels. Original files from confocal microscope in TIFF format are present as zipped Supplementary Information files. [file FEB4-12-95-s003.gif]
