# Supplementary material for: Accumulation of amyloid beta (Aβ) and amyloid precursor protein (APP) in tumors formed by a mouse xenograft model of inflammatory breast cancer
Source: FEBS Open Bio. 2021 Oct 26;12(1):95–105. doi: 10.1002/2211-5463.13308 (PMC8727955; doi:10.1002/2211-5463.13308)
Supplement: Supplementary file 2 — Fig. S2. DAPI staining of amyloid near blood vessels, Scale bar, 20 µm. [file FEB4-12-95-s001.pptx]

## Slide 1
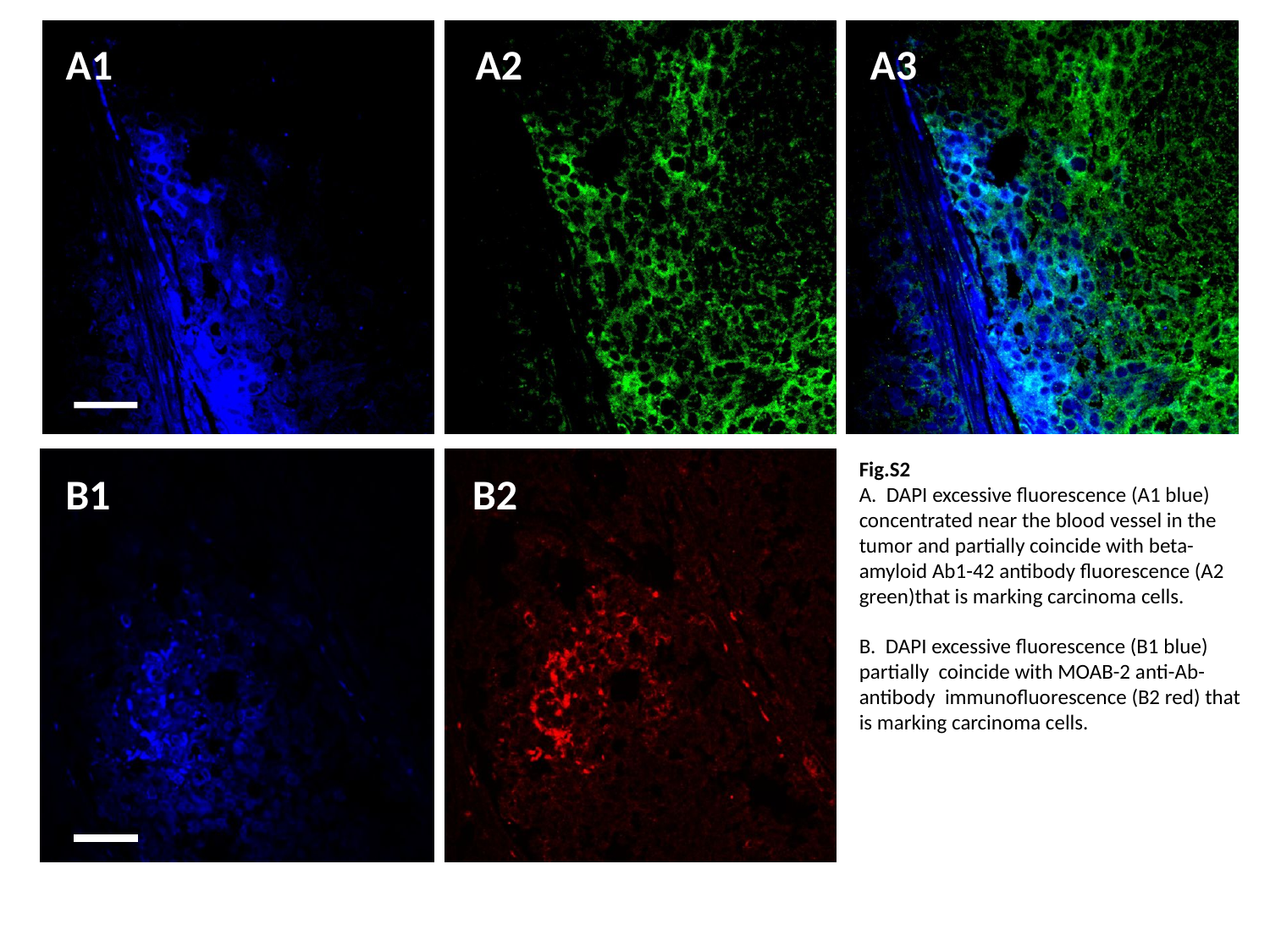

A1
A2
A3
Fig.S2
A. DAPI excessive fluorescence (A1 blue) concentrated near the blood vessel in the tumor and partially coincide with beta-amyloid Ab1-42 antibody fluorescence (A2 green)that is marking carcinoma cells.
B. DAPI excessive fluorescence (B1 blue) partially coincide with MOAB-2 anti-Ab-antibody immunofluorescence (B2 red) that is marking carcinoma cells.
B1
B2
